# Supplementary material for: Stress affects the epigenetic marks added by natural transposable element insertions in Drosophila melanogaster
Source: Sci Rep. 2018 Aug 15;8:12197. doi: 10.1038/s41598-018-30491-w (PMC6093896; doi:10.1038/s41598-018-30491-w)

Stress affects the epigenetic marks added by natural transposable element insertions in *Drosophila melanogaster*

Lain Guio, Cristina Vieira, and Josefa González

Supplementary Tables

Supplementary Table 1. List of DGRP lines used to create Outbred populations #2. All the lines used to create the outbred populations were obtained from The Drosophila Genetic Reference Panel project (DGRP)[21,22].

**Lines with *Bari-Jheh***	**Lines without *Bari-Jheh***
RAL-441	RAL-177
RAL-88	RAL-383
RAL-820	RAL-857
RAL-716	RAL-776
RAL-391	RAL-802
RAL-371	RAL-783
RAL-195	RAL-737

Supplementary Table 2. List of primers used in this study.

Gene	Primer	Sequence
Rp49 positive control for H3K4me3 [33]	rp49 F	cggatcgatatgctaagctgt
	rp49 R	gcgcttgttcgatccgta
18S positive control for H3K9me3 [32]	18S F	tttcatgcttgggattgtga
	18S R	gtacaaagggcagggacgta
Ultrabithorax (Ubx) positive control for H3K9me3 [34]	Ubx F	gaggcctgttcaaagtacgagt
	Ubx R	ggaaaccaattcgtgtgaaatc
Bari-CyP12a4	Bari-CyP-Fw	gcattgatcaacttgccaaa
	Bari-CyP-Rv	acttgctgcacaagcaactg
Bari-Ppcs	Bari-Ppcs-Fw	attcaggcaaatcggacaa
	Bari-Ppcs-Rv	tggctcaatggtatcacaaca
Bari-Jheh2	Bari-Jheh2 Fw	aacacgcggagcccttaata
	Bari-Jheh2 Rv	atttcaggcaaatcggacaa
Bari-Jheh3	Bari-Jheh3 Fw	caactgtttgtgacccatgc
	Bari-Jheh3 Rv	tgtttgtaattgaccgcaaa
Bari-Absent	Bari-Jheh2 Fw	aacacgcggagcccttaata
	Bari-Jheh3 Rv	tgtttgtaattgaccgcaaa
Jheh1 5’	Jheh1_Fw	cagtgacacccatctgttccta
	Jheh1-Rv	tatcatcatgagcgtcgaaaac
Jheh2 5’	Jheh2-Fw	gataaggttccttcaatgttggac
	Jheh2-Rv	gcataaacacacaatggcgaac
Jheh3 5’	Jheh3-Fw	aaagccgcttaagaactgactg
	Jheh3-Rv	atagccagcgctatctaaacga

Supplementary Table S3. p-values for control genes RpL32, 18SrRNA and Ubx comparing the enrichment level of the corresponding histone marks H3K4me3, H3K9me3 and H3K27me3 respectively, sort by condition. Corresponding to Supplementary Figure1 A), B) and C) respectively.

**Histone comparison**	**Condition**	**Gene**	**p-value**
H3K4me3 vs. H3K9me3	non-stress	RpL32	0.0001
H3K4me3 vs. H3K27me3	non-stress	RpL32	0.0001
H3K9me3 vs. H3K27me3	non-stress	RpL32	0.2507
H3K4me3 vs. H3K9me3	stress	RpL32	<0.0001
H3K4me3 vs. H3K27me3	stress	RpL32	0.0001
H3K9me3 vs. H3K27me3	stress	RpL32	0.3556
H3K4me3 vs. H3K9me3	non-stress	18SrRNA	<0.0001
H3K4me3 vs. H3K27me3	non-stress	18SrRNA	<0.0001
H3K9me3 vs. H3K27me3	non-stress	18SrRNA	0.2148
H3K4me3 vs. H3K9me3	stress	18SrRNA	<0.0001
H3K4me3 vs. H3K27me3	stress	18SrRNA	<0.0001
H3K9me3 vs. H3K27me3	stress	18SrRNA	0.7728
H3K4me3 vs. H3K9me3	non-stress	Ubx	0.6275
H3K4me3 vs. H3K27me3	non-stress	Ubx	<0.0001
H3K9me3 vs. H3K27me3	non-stress	Ubx	<0.0001
H3K4me3 vs. H3K9me3	stress	Ubx	0.5637
H3K4me3 vs. H3K27me3	stress	Ubx	<0.0001
H3K9me3 vs. H3K27me3	stress	Ubx	<0.0001

Supplementary Figures

Supplementary Figure S1. Testing immunoprecipitations and antibodies.

Enrichment of the histone marks relative to the input of each strain for each background under nonstress and stress conditions. Each panel represents a different genomic region well known to enrich a different histone mark: A) *18SrRNA* enriched in H3K9me3 and H3K27me3, B) *RpL32* enriched for H3K4me3 and C) *Ubx* enriched for H3K27me3.

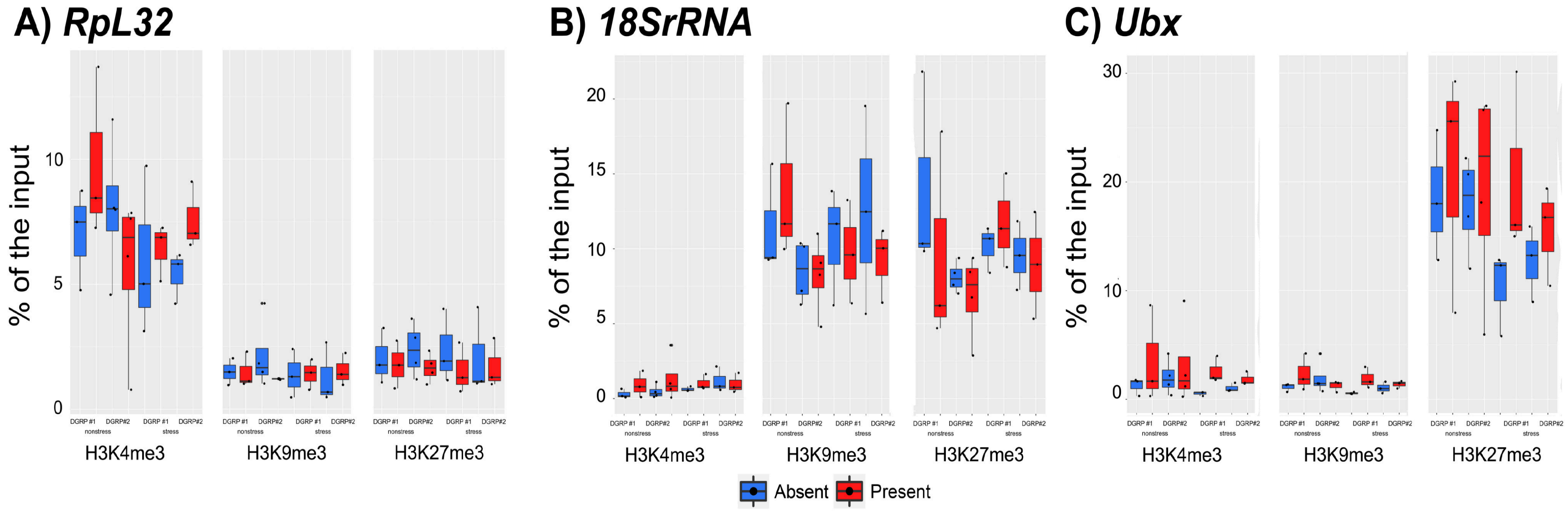

Supplement: Supplementary file 1 — Supplementary Information [file 41598_2018_30491_MOESM1_ESM.pdf]
